# Rainy-weather speed limit strategies on highways with variable longitudinal slopes

**DOI:** 10.1371/journal.pone.0354975

**Published:** 2026-07-30

**Authors:** Chunjie Li, Guanglin Sun, Xinjian Liu, Haiyuan Sun, Sen Qiu

**Affiliations:** 1 Hebei Expressway Group Limited, Shijiazhuang, China; 2 Research Institute for Road Safety of MPS, Beijing, China; 3 Guangzhou Fengyulei Technology Company Limited, Guangzhou, China; Shanghai University, CHINA

## Abstract

Rainfall substantially degrades highway traffic safety by reducing pavement friction, shortening driver perception distance, and amplifying the adverse effects of longitudinal grade on vehicle dynamics. To support refined speed management under rainy-weather conditions, this study develops a dynamic safe speed-limit model that jointly accounts for pavement friction, longitudinal slope, and perception distance. An improved Intelligent Driver Model (IDM) is further proposed to represent rainy-weather car-following behavior and speed adaptation. The proposed model was evaluated using the Hangzhou West-Fuxi highway section, and simulation experiments were conducted under different rainfall intensities and slope conditions. Model validation showed that the simulated speeds were consistent with observed speed characteristics, with RMSE values of 2.13–3.08 km/h and MAPE values of 2.31%−4.12%. The safety evaluation results indicated that the proposed speed-limit strategy reduced conflict rates by 7.1%, 73.6%, and 82.4% across the three rainfall scenarios compared with the unrestricted-speed condition. Sensitivity analysis further showed that increasing rainfall intensity reduced the pavement friction coefficient, increased braking distance, and narrowed the safety boundary, particularly on downhill sections. The results indicate that no additional speed restriction is generally required for rainfall intensities of 0–1.0 mm/min, whereas rainfall intensities of 1.0–5.0 mm/min require progressively stricter speed limits. A longitudinal slope of +1% provides the most favorable operating condition, while a −3% slope represents the most adverse case. These findings provide a quantitative basis for adaptive highway speed-limit control under rainy-weather conditions.

## Introduction

Rainfall is a major adverse weather factor affecting highway traffic safety and operational efficiency. Previous studies have frequently used historical weather, traffic, and crash records to identify crash-prone locations and predict accident risk under inclement weather conditions. In practice, traffic management agencies often adopt speed restrictions or traffic control measures to reduce weather-related risk. However, many existing control strategies are primarily applied to limited high-risk locations, while speed limits on mainline highway sections are often insufficiently differentiated with respect to local rainfall intensity, pavement condition, and geometric alignment. In addition, conventional control measures may suffer from delayed response, limited adaptability, and insufficient real-time information. Therefore, segment-level dynamic speed-limit strategies that respond to rainfall and roadway characteristics are needed to improve highway safety and traffic efficiency under rainy-weather conditions.

Vehicle speed control is a fundamental measure for improving roadway safety, especially in complex or high-risk environments such as tunnels [1–3], ramps [4], intersections [5], and steep grades [6]. Existing speed management approaches include enforcement-oriented measures using cameras and radar devices [7], in-vehicle assistance systems and speed-limit aids [8–10], and engineering countermeasures such as speed-limit signs [11], deceleration markings [12], speed bumps [13], and colored pavement [14]. Recent studies have further explored adaptive or intelligent speed control. For example, Li et al. proposed a closed-loop feedback speed guidance system that dynamically adjusts guidance strategies according to real-time vehicle operation and individual driving style to optimize ramp merging, reduce crash risk, and improve traffic efficiency [15]. Ge et al. investigated the influence of accompanying vehicle lighting length on driver behavior in long tunnel curves, focusing on speed regulation and steering accuracy at different speeds [16]. Li et al. examined dynamic illumination adjustment at highway intersection exits to improve nighttime safety and reduce energy consumption [17]. Ravani and Wang evaluated speeding problems in highway work zones and assessed the safety effectiveness of police presence strategies [18]. Bakibillah et al. developed ecological driving schemes that consider road curvature and surface conditions and use nonlinear model predictive control to optimize speed trajectories, thereby reducing fuel consumption and emissions [19,20].

Adverse weather conditions, including rain, snow, and fog, can alter visibility, pavement friction, and driver perception, thereby affecting car-following behavior, decision-making, comfort, and response capability [21–25]. Gao et al. reported that reduced visibility under hazy weather increases collision risk and changes car-following behavior by lengthening reaction time and reducing sensitivity to spacing variations [26]. Yasanthi and Mehran analyzed freeway free-flow speed variations under snowy conditions and highlighted the importance of frequent snow and ice removal [27]. Gaweesh and Ahmed evaluated a weather-based Variable Speed Limit (VSL) system on rural mountainous interstate highways in Wyoming using an Empirical Bayes before-after method and found significant crash reductions [28]. Das et al. examined how operating speed, roadway design, weather, and traffic volume jointly affect crash outcomes using datasets from Washington and Ohio [29]. Romanowska and Budzynski quantified the influence of adverse weather and time of day on expressway traffic flow using real-world data from Poland [30]. Yasanthi et al. proposed a Weather-Responsive Variable Speed Limit (WRVSL) framework that treats speed limits as weather-dependent random variables for rural highways under extreme weather conditions [31]. Guo et al. developed adverse-weather speed-limit functions by considering rainfall intensity, fog visibility, and snow-related road adhesion and optimized the functions using an adaptive genetic algorithm [32].

Although these studies provide important theoretical and practical foundations, several limitations remain. First, many weather-responsive speed-limit studies emphasize macroscopic traffic performance or crash reduction, whereas the coupled influence of rainfall intensity, pavement friction, longitudinal slope, and perception distance on safe speed limits has not been fully quantified. Second, simulation-based studies often evaluate safety using conflict indicators, but additional statistical validation against observed traffic characteristics is required to demonstrate model reliability. Third, the physical mechanism by which rainfall reduces friction and increases braking requirements should be explicitly examined to improve the interpretability and practical applicability of rainy-weather speed-limit models. Simulation provides a practical approach for evaluating rainy-weather traffic control strategies because it offers lower cost, higher repeatability, and greater safety than field experiments. Accordingly, this study develops a dynamic safe speed-limit method for highways under rainy-weather conditions and improves the IDM car-following model to capture speed adaptation under reduced friction and perception distance. The proposed model is validated using observed speed characteristics through RMSE and MAPE, while safety effectiveness is evaluated using Time-to-Collision (TTC)-based conflict rates. In addition, a sensitivity analysis of rainfall intensity, pavement friction coefficient, and braking distance is conducted to clarify the physical basis of the proposed speed-limit strategy. The main contributions of this study are summarized as follows:

- A rainy-weather dynamic safe speed-limit model is established by integrating pavement friction, longitudinal slope, and driver perception distance into a unified safety constraint.
- An improved IDM car-following model is developed and validated using highway simulation and observed speed characteristics to evaluate the effectiveness of the proposed rainy-weather speed-limit strategy.

## Materials and methods

### Establishment of the safe speed limit model

The determination of the safe speed limit is primarily influenced by factors such as the friction coefficient, road gradient, and perception distance. These parameters should be comprehensively considered when analyzing the safe following distance.

To ensure driving safety, the following distance between vehicles must satisfy the safe driving condition. The total distance traveled by the following vehicle—including the distance covered during the driver’s reaction time *S*_*t*_, the braking distance after deceleration *S*_*d*_, the static safety distance *S*_*s*_, and the length of the preceding vehicle *S*_*l*_—must not exceed the sum of the initial spacing between the two vehicles *S*_*n*_ and the distance traveled by the leading vehicle during the same period *S*_*m*_. The mathematical expression of this safe driving condition is given in Equation 1.


$$\begin{array}{c} S_{t}+S_{d}+S_{s}+S_{l}⩽S_{n}+S_{m} \end{array}$$ (1)

In Equation 1, *S*_*t*_, *S*_*d*_, *S*_*n*_, and *S*_*m*_ are variables, whereas *S*_*s*_ and *S*_*l*_ are constants. The formulations for *S*_*t*_ and *S*_*d*_ are presented in Equations 2 and 3, respectively. Equation 2 defines the reaction distance, which is the distance traveled by the following vehicle at a constant speed during the driver’s reaction time after detecting the leading vehicle or an obstacle. Equation 3 defines the braking distance, referring to the distance the vehicle covers from the initiation of braking until it comes to a complete stop.


$$\begin{array}{c} S_{t}=\frac{v\cdot t_{r}}{\text{3}.\text{6}} \end{array}$$ (2)

$$\begin{array}{c} S_{d}=\frac{(v/\text{3}.\text{6}{)}^{\text{2}}}{\text{2}g(f\pm i)} \end{array}$$ (3)

In the equations, *v* denotes the speed of the following vehicle; *t*_*r*_ represents the reaction time, which includes the response delay of the onboard sensors and control systems. Under rainy conditions, *t*_*r*_ is assumed to be 2.5s to reflect the most adverse scenario. *f* is the pavement friction coefficient, *i* represents the longitudinal grade of the roadway (positive for uphill and negative for downhill), and *g* denotes the gravitational acceleration, typically taken as 9.8m/s^2^.

To account for the most unfavorable driving conditions in rainy weather, *S*_*m*_ is set to zero. The safe driving condition can thus be expressed as shown in Equation 4. When the spacing between vehicles is defined as the perception distance and the critical state of safe driving is considered, the corresponding vehicle speed represents the *maximum safe speed*. Based on Equation 4, Equation 5 is derived to describe the relationship between perception distance and the safe speed limit under safety constraints.


$$\begin{array}{c} \frac{v\cdot t_{r}}{\text{3}.\text{6}}+\frac{v^{\text{2}}}{\text{2}\text{5}\text{4}(f\pm i)}+S_{s}+S_{l}⩽S_{n} \end{array}$$ (4)

$$\begin{array}{c} \frac{v_{safe}\cdot t_{r}}{\text{3}.\text{6}}+\frac{v_{safe}^{\text{2}}}{\text{2}\text{5}\text{4}(f\pm i)}+S_{s}+S_{l}⩽S_{c} \end{array}$$ (5)

In the equations, *v*_*safe*_ represents the safe speed limit, and *S*_*c*_ denotes the vehicle’s perception distance. The safe speed limit is primarily influenced by the friction coefficient, road gradient, and perception distance.

Under rainy conditions, both the friction coefficient and perception distance vary with rainfall intensity. Specifically, during light or moderate rain, the friction coefficient is mainly affected by vehicle speed. In contrast, during heavy or torrential rain, both vehicle speed and the thickness of the water film must be considered, as they jointly affect the friction coefficient. Equation 6 provides the expression for the friction coefficient under light or moderate rain, Equation 7 defines the corresponding expression under heavy or torrential rain, and Equation 8 presents the formulation for water film thickness used in Equation 7.


$$\begin{array}{c} f=\text{2}.\text{3}\text{5}\times \text{1}{\text{0}}^{-\text{5}}v^{\text{2}}-\text{4}.\text{5}\text{1}\text{8}\times \text{1}{\text{0}}^{-\text{3}}v+\text{0}.\text{5}\text{0}\text{8}\text{6},q\in Q_{\text{1}} \end{array}$$ (6)

$$\begin{array}{c} f=\text{0}.\text{9}\text{4}\text{5}\text{8}-\text{0}.\text{0}\text{0}\text{5}\text{7}v-\text{0}.\text{0}\text{1}\text{1}\text{8}h,q\in Q_{\text{2}} \end{array}$$ (7)

$$\begin{array}{c} h=\text{1}.\text{3}\text{5}\text{8}\text{9}{\left[\frac{q\cdot n\cdot l_{x}\cdot {\left(i_{x}^{\text{2}}+i_{y}^{\text{2}}\right)}^{\text{1}/\text{4}}}{i_{x}}\right]}^{\text{3}/\text{5}} \end{array}$$ (8)

In the equations, *h* represents the water film thickness; *q* denotes the rainfall intensity; *n* is the surface roughness coefficient, taken as 0.016 for asphalt pavement; *l*_*x*_ refers to the transverse slope length; *i*_*x*_ represents the transverse slope of the pavement; *i*_*y*_ denotes the longitudinal slope; *Q*_1_ corresponds to the set of rainfall intensities for light or moderate rain; and *Q*_2_ corresponds to those for heavy or torrential rain.

The vehicle’s perception distance depends on the driver’s visible range. In this study, the perception distance is set to 250 m under normal weather conditions. To ensure safety, it is reduced to 200 m during light or moderate rainfall. Under heavy or torrential rain, the perception distance is determined by the meteorological visibility provided by the meteorological authority and is generally set to 47.5 m.

**Fig 1:**
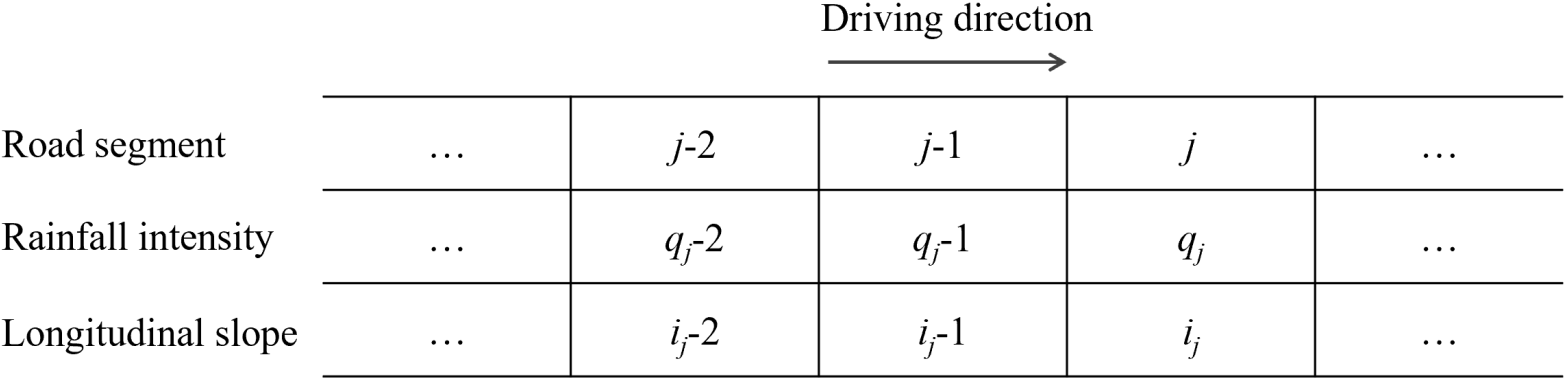
Schematic of speed limits for different road segments under rainy conditions.

To enable refined management of highway speed limits during rainfall, this study proposes a dynamic safe speed limit method that determines appropriate speed limits for different road segments based on real-time rainfall intensity and longitudinal slope. The highway is divided into segments according to slope gradients, as illustrated in Fig 1.

The procedure for determining the speed limit for each road segment is as follows. First, the safe speed limit for each segment is calculated using Equation 5. Next, using the segment with the lowest speed limit as a reference, the speed limits for upstream and downstream segments are determined sequentially. Finally, all segment speed limits are adjusted in accordance with relevant regulations. Specifically, each segment’s speed limit should be rounded to the nearest multiple of 10 km/h, the difference in speed limits between adjacent segments should not exceed 20 km/h, and the maximum speed limit is capped at 120 km/h. Equation 9 represents the safe speed limit for the segment with the lowest speed, while Equations 10–12 provide the method for calculating the safe speed limit for segment *j*.


$$\begin{array}{c} V_{m}=\text{min}\left(V_{\text{max}},V_{m,safe}\right) \end{array}$$ (9)

$$\begin{array}{c} V_{j}=\text{min}\left(V_{\text{max}},V_{j,safe},V_{j-\text{1}}+p_{j}\right) \end{array}$$ (10)

$$\begin{array}{c} k_{j}=V_{j}-V_{j-\text{1}} \end{array}$$ (11)

$$\begin{array}{c} p_{j}=\left\{ \begin{array}{c} @c\text{0},\\ \text{1}\text{0}k_{j}/\left|k_{j}\right|,\\ \text{2}\text{0}k_{j}/\left|k_{j}\right|, \end{array}\begin{array}{c} \forall \left|k_{j}\right|<\text{1}\text{0}\\ \forall \text{1}\text{0}⩽\left|k_{j}\right|<\text{2}\text{0}\\ \forall \text{2}\text{0}⩽\left|k_{j}\right| \end{array}\right. \end{array}$$ (12)

In the equations, *V*_*m*_ represents the speed limit of the segment with the lowest speed, while *V*_*max*_ denotes the design speed of the segment. *V*_*m, safe*_ is the safe speed limit for the lowest-speed segment under rainy conditions, calculated using Equation 5. *V*_*j*_ refers to the speed limit of segment *j*, and *V*_*j, safe*_ is its corresponding safe speed limit under rainy conditions, also derived from Equation 5. *V*_*j*-1_ indicates the speed limit of the preceding segment *j*-1. *p*_*j*_ is a multiple of 10 that represents the actual speed difference between adjacent segments, with permissible values *p*_*j*_ ∈ {0, ±10, ±20}. Finally, *k*_*j*_ denotes the difference in safe speed limits under rainy conditions between adjacent segments.

### Enhancement of the car-following model based on safe speed limits

To simulate the influence of rainy weather on vehicle car-following behavior, this study proposes an enhancement of the Intelligent Driver Model (IDM). For clarity and consistency in the subsequent analysis, the following assumptions are made regarding vehicle characteristics: all vehicles have identical reaction times, measurement errors are neglected, and all vehicles are standard passenger cars.

(1) Car-Following Model Enhancement

The IDM is fundamentally an acceleration-based model, and its formulation is presented in Equations 13–16:


$$\begin{array}{c} {\dot{v}}_{\alpha }(t)=a^{\left(\alpha \right)}\left[\text{1}-{\left(\frac{v_{\alpha }(t)}{v_{\text{0}}^{\left(\alpha \right)}}\right)}^{\delta }-{\left(\frac{s^{*}\left(v_{\alpha }(t),\Delta v_{\alpha }(t)\right)}{s_{\alpha }(t)}\right)}^{\text{2}}\right] \end{array}$$ (13)

$$\begin{array}{c} s^{*}\left(v(t),\Delta v(t)\right)=s_{\text{0}}^{\alpha }+s_{\text{1}}^{\left(\alpha \right)}\sqrt{\frac{v(t)}{v_{\text{0}}^{\left(\alpha \right)}}}+T^{\alpha }v(t)+\frac{v(t)\cdot \Delta v(t)}{\text{2}\sqrt{a^{\left(\alpha \right)}\cdot b^{\left(\alpha \right)}}} \end{array}$$ (14)

$$\begin{array}{c} \Delta v_{\alpha }(t)=v_{\alpha }(t)-v_{\alpha -\text{1}}(t) \end{array}$$ (15)

$$\begin{array}{c} s_{\alpha }(t)=X_{\alpha -\text{1}}(t)-X_{\alpha }(t)-l_{\alpha } \end{array}$$ (16)

In the equations, ${\dot{v}}_{\alpha }$ represents the acceleration of vehicle *α*; *a*^(*α*)^ denotes its maximum acceleration; *δ* is the acceleration exponent; *v*_*α*_ is the actual speed of vehicle *α*; $v_{\text{0}}^{\alpha }$ is its desired speed; and *Δv*_*α*_ represents the speed difference between vehicle α and the preceding vehicle. s^*^ denotes the desired spacing, *s*_*α*_ is the actual distance between vehicle *α* and the vehicle ahead, $s_{\text{0}}^{\alpha }$ is the standstill safety distance, and $s_{\text{1}}^{\alpha }$ is the speed-dependent safety distance parameter. *T*^*α*^ represents the safe time headway, *b*^(*α*)^ is the comfortable deceleration, *x*_*α*-1_ and *x*_*α*_ are the longitudinal positions of the leading vehicle *α*-1 and the following vehicle *α*, respectively, and *l*_*α*_ is the vehicle length of *α*.

Under rainy conditions, several IDM parameters—including the desired speed, maximum acceleration, comfortable deceleration, and safe time headway—are affected by rainfall intensity. Accordingly, the IDM car-following model must be adapted to account for these changes. Equations 17–18 present the primary expressions of the improved car-following model, while Equation 19 defines the desired speed under rainy conditions, which is determined by both the road speed limit and the safe speed limit for rainy weather.


$$\begin{array}{c} {\dot{v}}_{\alpha }(t)=a_{r}^{\left(\alpha \right)}\left[\text{1}-{\left(\frac{v_{\alpha }(t)}{v_{\text{0}}^{\left(\alpha \right)}}\right)}^{\delta }-{\left(\frac{s^{*}\left(v_{\alpha }(t),\Delta v_{\alpha }(t)\right)}{s_{\alpha }(t)}\right)}^{\text{2}}\right] \end{array}$$ (17)

$$\begin{array}{c} s^{*}\left(v(t),\Delta v(t)\right)=s_{\text{0}}^{\alpha }+s_{\text{1}}^{\left(\alpha \right)}\sqrt{\frac{v(t)}{v_{des}^{\left(\alpha \right)}}}+T_{r}^{\alpha }\cdot v(t)+\frac{v(t)\cdot \Delta v(t)}{\text{2}\sqrt{a^{\left(\alpha \right)}\cdot b_{r}^{\left(\alpha \right)}}} \end{array}$$ (18)

$$\begin{array}{c} v_{des}=\text{min}\left(v_{\text{max}},v_{safe}\right) \end{array}$$ (19)

In the equations, $a_{r}^{\left(\alpha \right)}$ represents the maximum acceleration of vehicle *α* under rainy conditions; $v_{des}^{\left(\alpha \right)}$ denotes its desired speed in rain; $T_{r}^{\alpha }$ is the safe time headway between vehicle *α* and the preceding vehicle under rainy conditions; $b_{r}^{\left(\alpha \right)}$ indicates the comfortable deceleration of vehicle *α* in rain; and *V*_*max*_ corresponds to the road speed limit, i.e., the design speed.

(2) Desired Speed Optimization in the Improved Car-Following Model

To maintain optimal vehicle speed under rainy conditions, this study proposes a desired speed optimization method based on time headway. Vehicle motion states are classified according to time headway, with two critical thresholds: the free-driving critical time headway *t*_*max*_ and the braking critical time headway *t*_*min*_. Specifically:

1. Free-driving state: If the time headway *t* > *t*_*max*_, *t*he vehicle is considered to be in a free-driving state.
2. Car-following state: If *t*_*min*_ ≤ *t* ≤ *t*_*max*_, the vehicle is in a car-following state and aims to maintain the desired time headway *t*_*des*_ to achieve optimal speed.
3. Braking state: If *t* < *t*_*min*_, *t*he vehicle enters a braking state.

**Fig 2:**
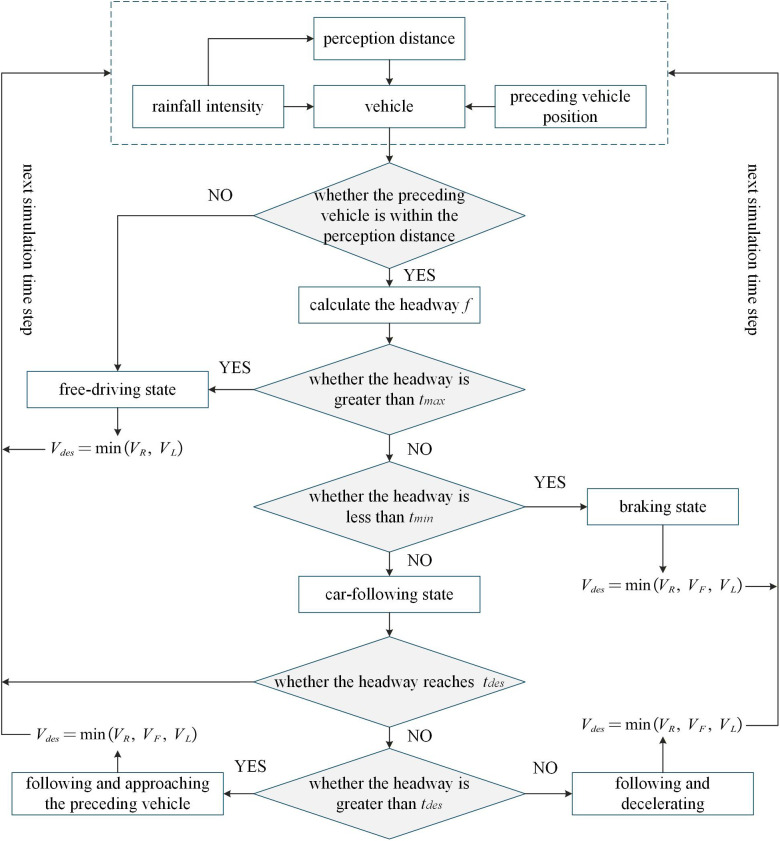
Process for desired speed optimization.

As illustrated in Fig 2, the optimization process under rainy conditions is conducted as follows:

1. The vehicle detects the preceding vehicle based on rainfall intensity and the corresponding perception distance. If the preceding vehicle is outside the perception range, the vehicle remains in a free-driving state, and the desired speed is set to the minimum of *V*_*R*_ and *V*_*L*_, corresponding to *V*_*safe*_ and *V*_*max*_, respectively. If the preceding vehicle is within the perception range, the time headway t is calculated.
2. Comparison with *t*_*max*_: If *t* > *t*_*max*_, *t*he vehicle remains in a free-driving state. If *t* ≤ *t*_*max*_, *t*he vehicle’s headway is further compared with *t*_*min*_.
3. Comparison with *t*_*min*_: If *t* < *t*_*min*_, *t*he vehicle enters a braking state, and the desired speed is set to the minimum of *V*_*R*_, *V*_*L*_, and *V*_*F*_, where *V*_*F*_ is introduced to enforce deceleration (see Equation 20). If *t* ≥ *t*_*min*_, *t*he vehicle is in a car-following state.
4. Car-following adjustment: While in the car-following state, if *t* ≥ *t*_*des*_, the vehicle maintains its current speed for the next calculation step. If *t* < *t*_*des*_, *t*he desired speed is adjusted to the minimum of *V*_*R*_, *V*_*F*_, and *V*_*L*_.

This method allows vehicles to dynamically adjust their speed according to both headway and rainy conditions, ensuring safety while optimizing traffic flow.


$$\begin{array}{c} V_{F}=\left\{ \begin{array}{c} \text{max}\left(V_{fo},V_{ld}\right),t>t_{des}\\ \text{min}\left(V_{fo},V_{ld}/\text{2}\right),t<t_{des} \end{array}\right. \end{array}$$ (20)

In the equation, *V*_*fo*_ represents the velocity of the following vehicle, whereas *V*_*ld*_ denotes the velocity of the leading vehicle.

### Driving simulation experiment

The experiment is conducted on the Hangzhou West–Fuxi section of the TJRD TS dataset, representing a highway mainline. This segment is 1.5 km long, with a design speed of 100 km/h, and consists of six lanes in each direction, each 3.75 m wide. All horizontal curves have radii exceeding 1000 m, allowing the segment to be approximated as a straight road. Based on the longitudinal slope, the section is divided into three subsegments, with their respective lengths and slopes summarized in Table 1.

**Table 1:** Segment lengths and longitudinal slopes of the Hangzhou West–Fuxi highway detection section.

Title	Section 1	Section 2	Section 3
midpoint chainage	K95 + 618	K96 + 132	K96 + 637
length (km)	0.490	0.537	0.473
curve radius (m)	6000	1600	1800
longitudinal slope (%)	−0. 90	−2.50	−1.80

In the simulation, the transverse slope is set to 0.02%, the total simulation time is 4000 s, and a 400 s warm-up period is used. To replicate rapid changes in rainfall, the rainfall intensity for the three segments is set to increase sequentially, denoted as q1, q2, and q3. Using the rainy-weather highway speed limit method, the speed limits for each segment are calculated, resulting in *V*_1_, *V*_2_, and *V*_3_, as listed in Table 2.

**Table 2:** Corresponding speed limits under varying rainfall intensities in the simulation experiment.

Title	rainfall intensity (mm/min)			speed limit (km/h)		
	*q*_1_	*q*_2_	*q*_3_	*v*_1_	*v*_2_	*v*_3_
test 1	0.9	1.0	1.1	100	100	90
test 2	1.4	1.5	1.6	80	60	60
test 3	1.9	2.0	2.1	60	60	60

The driving simulation scenarios were constructed in UC-win/Road based on imported road BIM (Building Information Modeling) models and include three rainfall intensity levels.

## Results

### Statistical validation

To quantitatively evaluate the reliability of the simulation model, the simulated traffic performance was compared with observed traffic data from the Hangzhou West-Fuxi highway section. Root mean square error (RMSE) and mean absolute percentage error (MAPE) were adopted to evaluate the model’s ability to reproduce observed speed characteristics under different rainfall intensities.

Because the simulation traffic demand was set according to the observed traffic volume, the GEH statistic cannot be used as an independent validation indicator in this study. Therefore, this paper focuses on RMSE and MAPE for speed validation rather than using GEH for traffic-volume validation.

As shown in Table 3, the RMSE values of the simulated speeds ranged from 2.13 to 3.08 km/h, and the MAPE values ranged from 2.31% to 4.12%, indicating that the simulated speeds were close to the observed speeds under different rainfall intensities. These results demonstrate that the improved IDM model can reasonably reproduce rainy-weather traffic operating conditions.

**Table 3:** Statistical validation results of the simulation model.

Scenario	Rainfall intensity (mm/min)	Observed mean speed (km/h)	Simulated mean speed (km/h)	Speed error (km/h)	RMSE (km/h)	MAPE (%)
Test 1	0.9-1.1	92.5	90.8	−1.7	2.13	2.31
Test 2	1.4-1.6	74.2	71.8	−2.4	2.86	3.64
Test 3	1.9-2.1	63.6	61.1	−2.5	3.08	4.12

**Fig 3:**
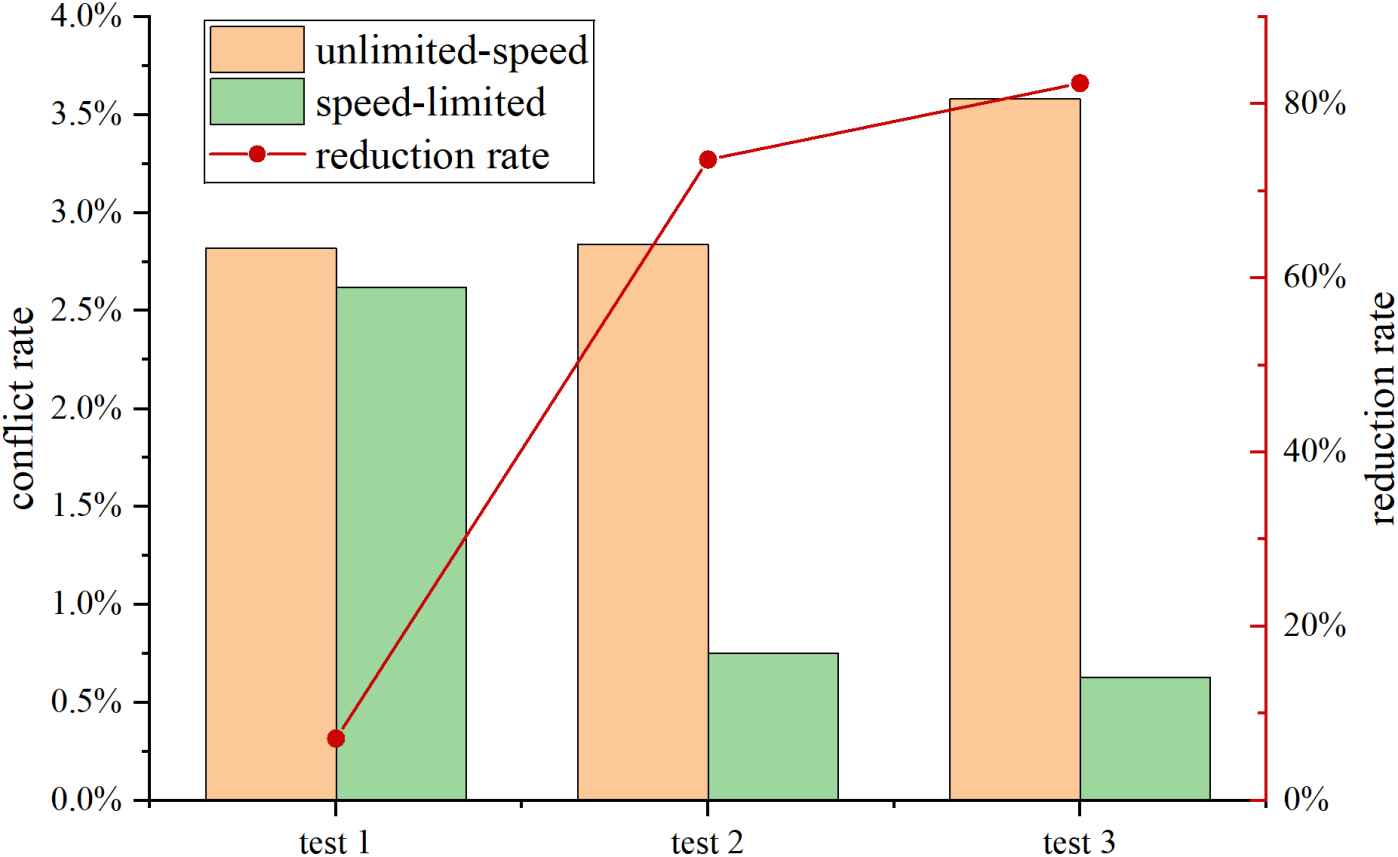
Comparison of conflict rates.

In the simulation experiments, the total number of conflicts across all time steps was recorded to compare traffic conflicts and conflict rates under two scenarios: rainy-weather speed limits and unrestricted speed. Time-to-collision (TTC) was adopted as the conflict indicator, and the results are summarized in Table 4. Statistical analysis indicates that, across the three experimental scenarios, the conflict rates under the proposed speed-limit scheme were reduced by 7.1%, 73.6%, and 82.4%, respectively, compared with the unrestricted-speed condition (Table 5 and Fig 3). These results demonstrate that the proposed rainy-weather highway speed-limit method is highly effective, substantially reducing both the number of conflicts and conflict rates, and thereby mitigating highway accidents under rainy conditions.

**Table 4:** Comparison of simulation results between the unlimited-speed and speed-limited scenarios.

Title	Number of conflicts		Conflict rate (%)	
	Unlimited-speed	Speed-limited	Unlimited-speed	Speed-limited
**test 1**	14408	13436	2.82	2.62
**test 2**	14532	4396	2.84	0.75
**test 3**	18724	3848	3.58	0.63

**Table 5:** Analysis and comparison of conflict rates.

Title	Conflict rate (%)		Reduction rate
	Unlimited-speed	Speed-limited	
**test 1**	2.82	2.62	7.1%
**test 2**	2.84	0.75	73.6%
**test 3**	3.58	0.63	82.4%

### Sensitivity analysis

To further clarify the physical influence of rainfall intensity on vehicle dynamics, a sensitivity analysis was conducted based on Equations (6)–(8). Rainfall intensity was varied from 1.0 to 5.0 mm/min, representative longitudinal slopes of -3%, -1%, 0%, 1%, and 3% were considered, and the vehicle speed was fixed at 100 km/h. The braking distance was calculated using the safe-distance model, and the change rate was calculated relative to the braking distance at 1.0 mm/min under the same longitudinal slope.

As shown in Table 6, as rainfall intensity increased from 1.0 to 5.0 mm/min, the pavement friction coefficient decreased from approximately 0.373 to 0.357. Under the same rainfall intensity, downhill sections produced longer braking distances than uphill sections. For example, at 5.0 mm/min, the braking distance at a -3% grade reached approximately 121.6 m, whereas that at a +3% grade was approximately 102.3 m. This indicates that increasing rainfall intensity reduces tire-pavement adhesion, lengthens braking distance, narrows the safety boundary, and consequently requires lower safe speed limits under heavy rainfall conditions.

**Table 6:** Sensitivity analysis of rainfall intensity, friction coefficient, and braking distance.

Rainfall intensity (mm/min)	Longitudinal slope (%)	Friction coefficient	Braking distance at 100 km/h (m)	Change in braking distance (%)
1.0	−3	0.373	114.9	0.0
1.5	−3	0.371	115.7	0.7
2.0	−3	0.369	116.5	1.4
3.0	−3	0.365	118.1	2.8
4.0	−3	0.361	119.8	4.3
5.0	−3	0.357	121.6	5.8
1.0	−1	0.373	108.3	0.0
1.5	−1	0.371	109.0	0.6
2.0	−1	0.369	109.7	1.3
3.0	−1	0.365	111.0	2.5
4.0	−1	0.361	112.5	3.9
5.0	−1	0.357	113.9	5.2
1.0	0	0.373	105.5	0.0
1.5	0	0.371	106.1	0.6
2.0	0	0.369	106.8	1.2
3.0	0	0.365	108.0	2.4
4.0	0	0.361	109.3	3.6
5.0	0	0.357	110.5	4.7
1.0	1	0.373	102.7	0.0
1.5	1	0.371	103.4	0.7
2.0	1	0.369	104.0	1.3
3.0	1	0.365	105.2	2.4
4.0	1	0.361	106.4	3.6
5.0	1	0.357	107.6	4.8
1.0	3	0.373	97.6	0.0
1.5	3	0.371	98.2	0.6
2.0	3	0.369	98.8	1.2
3.0	3	0.365	99.9	2.4
4.0	3	0.361	101.1	3.6
5.0	3	0.357	102.3	4.8

**Fig 4:**
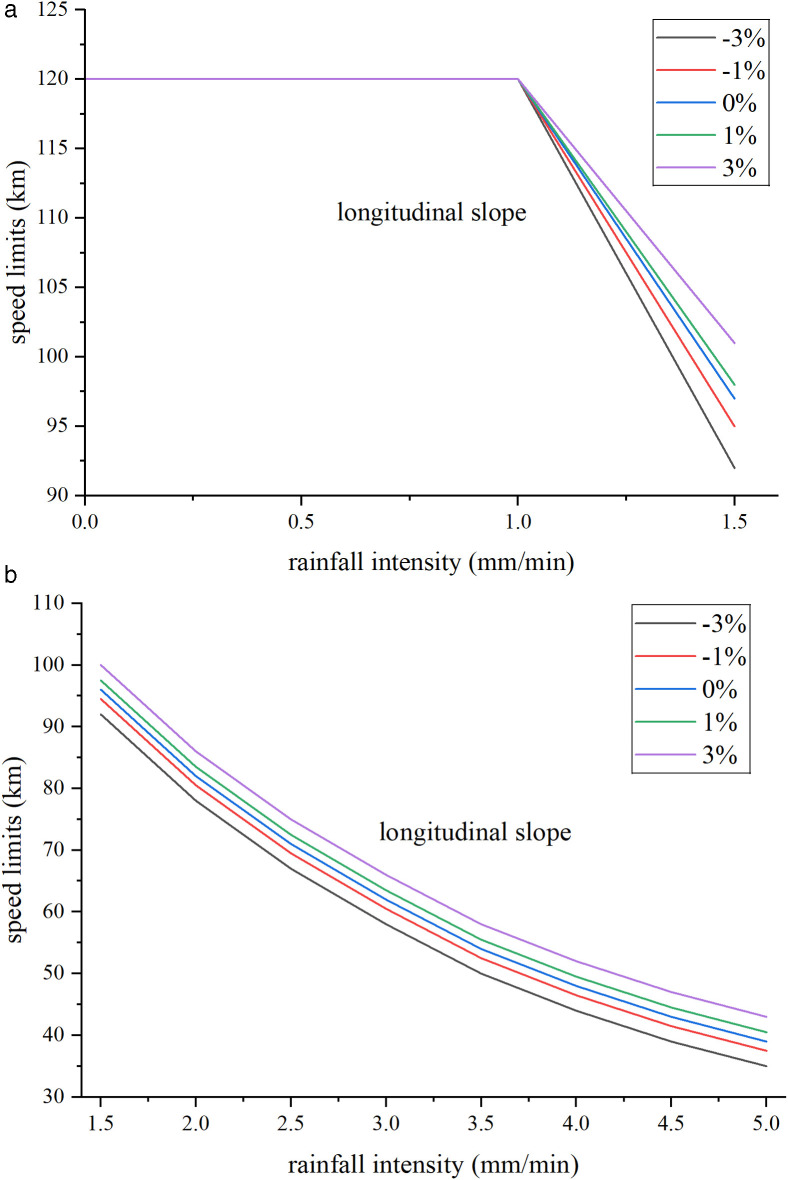
Safe speed limits for different road grades and varying rainfall intensities. (a) Safe speed limits under light/moderate rain conditions. (b) Safe speed limits under heavy/extreme rain conditions.

To further examine appropriate speed limit settings under varying longitudinal slopes and rainfall intensities, a simulation scenario was developed for a highway with a design speed of 120 km/h. Based on rainfall intensity categories, safety speed limit curves were plotted for different longitudinal slopes and rainfall intensities, as shown in Fig 4. The analysis revealed the following:

1. As rainfall intensity increases, the vehicle speed limit generally decreases. Within the range of (0, 1.0] mm/min, the speed limit can remain at 120 km/h. In the range of (1.0, 5.0] mm/min, the speed limit decreases progressively, with the sub-range (1.5, 5.0] showing more pronounced reductions than the (1.0, 1.5] sub-range.
2. The effect of longitudinal slope on the safety speed limit follows a consistent trend as rainfall intensity increases. A longitudinal slope of 1% allows vehicles to maintain the highest safe speed, representing optimal driving conditions, whereas a slope of −3% corresponds to the lowest safe speed, indicating the most adverse driving conditions.

## Discussion

The results of this study highlight the crucial role of dynamic speed limits in enhancing both vehicle safety and traffic efficiency, particularly under adverse weather conditions such as rainfall. As rainfall intensity increases, the risk of traffic conflicts escalates, making the implementation of adaptive speed limits essential for mitigating potential hazards. Our findings demonstrate that the proposed dynamic speed limit model, which incorporates factors such as road gradient, friction coefficient, and perception distance, provides a more reliable and efficient approach to managing traffic flow during rainy conditions.

One notable advantage of simulation-based research, as shown in this study, is its cost-effectiveness and high repeatability compared to real-world testing. Simulations allow for the controlled examination of various traffic scenarios, road conditions, and weather intensities without the risks and constraints associated with actual field experiments. Moreover, the dynamic speed limit method proposed here offers real-time adaptability, adjusting speed limits according to changing weather conditions, thereby increasing its practical applicability in real-world traffic management systems.

However, several areas warrant further investigation. For example, the model could be extended to incorporate additional variables, such as wind speed, visibility, and vehicle types, all of which affect safe driving speeds in adverse weather. Future research could also assess the effectiveness of this dynamic speed limit system across different geographic regions, considering variations in road conditions and weather patterns.

The implications of this study are significant, particularly for regions frequently experiencing heavy rainfall or other severe weather conditions. By adopting simulation-based approaches, traffic authorities can develop more adaptive and responsive traffic management systems, improving road safety while also enhancing traffic flow efficiency, reducing congestion, and preventing accidents.

## Conclusions

This study begins by analyzing the mechanisms through which rainfall affects vehicle safety. Key factors such as the friction coefficient, road gradient, and perception distance are considered to develop a safety speed limit model for rainy conditions. A dynamic speed limit method is also proposed. Next, the IDM (Intelligent Driver Model) car-following model is improved to account for rainy conditions, and a new car-following model for highways is introduced. The validity of the proposed speed limit method and the modified car-following model is verified using the Hangzhou Xi-Fu Expressway as an experimental scenario. Finally, a highway simulation environment is created to explore the establishment of reasonable speed limits under different road gradients and rainfall intensities. The results indicate that when rainfall intensity is between (0, 1.0] mm/min, no speed reduction is necessary. However, when rainfall intensity ranges from (1.0, 5.0] mm/min, a reasonable speed limit should be enforced. As rainfall intensity increases, the influence of different road gradients on the marginal strength of the safety speed limit remains consistent. Specifically, when the road gradient is +1%, vehicles can maintain the highest safe speed, representing optimal driving conditions, while at a -3% gradient, the safe speed limit is minimized, representing the least favorable driving conditions.

## References

[1] Wan H, Du Z, Ran B, Wang M. Speed control method for highway tunnel safety based on visual illusion. Transp Res Rec: J Transp Res Board. 2015;2485(1):1–7. doi: 10.3141/2485-01

[2] Chen Y, Du Z, Xu J, Luo S. Impact of traffic signs on driving speed at mountain highway tunnel entrances − the role of low-volume intermittent information. Transp Res Part F: Traffic Psychol Behav. 2024;106:328–39. doi: 10.1016/j.trf.2024.08.021

[3] Wang X, Feng Y, Fan Y, Lian Z, Cao J, Wang H. A field implementation of traffic speed harmonisation on the highway with long-tunnel clusters. Transportmetrica A: Transp Sci. 2025;:1–31. doi: 10.1080/23249935.2025.2454251

[4] Zhu J, Easa S, Gao K. Merging control strategies of connected and autonomous vehicles at freeway on-ramps: a comprehensive review. J Intell Connect Vehic. 2022;5(2):99–111. doi: 10.1108/jicv-02-2022-0005

[5] Wan H, Chen X, Du Z. Improving safety and efficiency of roundabouts through an integrated system of guide signs. Sustainability. 2019;11(19):5202. doi: 10.3390/su11195202

[6] Wu Y, Qiao J, Shekonya KP, Qiao J. Speed control and driver psychophysiology performance in highway uphill sections. Transp Res Rec: J Transp Res Board. 2023;2677(8):347–60. doi: 10.1177/03611981231156590

[7] Shi K, He S, Shi Z, Chen A, Xiong Z, Chen J, et al. Radar and camera fusion for object detection and tracking: a comprehensive survey. IEEE Commun Surv Tutorials. 2026;28:3478–520. doi: 10.1109/comst.2025.3599596

[8] Ayyasamy S. A comprehensive review on advanced driver assistance system. J Soft Comput Paradigm. 2022;4(2):69–81. doi: 10.36548/jscp.2022.2.003

[9] Ma Z, Zhang Y. Drivers trust, acceptance, and takeover behaviors in fully automated vehicles: effects of automated driving styles and driver’s driving styles. Accid Anal Prev. 2021;159:106238. doi: 10.1016/j.aap.2021.106238 34182321

[10] Lu Y, Yi B, Song X, Zhao S, Wang J, Cao H. Can we adapt to highly automated vehicles as passengers? The mediating effect of trust and situational awareness on role adaption moderated by automated driving style. Transp Res Part F: Traffic Psychol Behav. 2022;90:269–86. doi: 10.1016/j.trf.2022.08.011

[11] Bellini D, Iaconis MC, Traettino E. Speed limits and road warning signs as aid for driving behavior. Transp Res Procedia. 2020;45:135–42. doi: 10.1016/j.trpro.2020.02.100

[12] Park N, Park S, Park J, Kim M. Evaluation of vehicle deceleration effects for peripheral transverse line markings on urban roads. J Transp Eng, Part A: Systems. 2023;149(12). doi: 10.1061/jtepbs.teeng-7980

[13] Lav AH, Bilgin E, Lav AH. A fundamental experimental approach for optimal design of speed bumps. Accid Anal Prev. 2018;116:53–68. doi: 10.1016/j.aap.2017.05.022 28583281

[14] Wang K, Gudyanga B, Zhang W, Feng Z, Wang C, Yang B, et al. Optimization of colored pavement considering driving behavior and psychological characteristics under dynamic low-visibility conditions related to fog-a driving simulator study. Traffic Inj Prev. 2024;25(3):518–26. doi: 10.1080/15389588.2024.2308523 38346171

[15] Li H, Xiao T, Li Y, Feng Y. Development and safety evaluation of an adaptive personalized speed guidance system for on-ramp merging in highway service areas. Transp Res Part A: Policy Pract. 2024;190:104296. doi: 10.1016/j.tra.2024.104296

[16] Ge H, Song C, Jing D, Li W, Shi Z, Guo Z, et al. Analysis of the effect of highway extra-long tunnel Accompanying vehicle lighting on driving behavior in horizontal curve − a driving simulation study. Tunnell Underground Space Technol. 2025;157:106286. doi: 10.1016/j.tust.2024.106286

[17] Li H, Wang L, Yang M, Bie Y. Collaborative effects of vehicle speed and illumination gradient at highway intersection exits on drivers’ stress response capacity. Accid Anal Prev. 2025;209:107829. doi: 10.1016/j.aap.2024.107829 39488858

[18] Ravani B, Wang C. Speeding in highway work zone: an evaluation of methods of speed control. Accid Anal Prev. 2018;113:202–12. doi: 10.1016/j.aap.2018.01.030 29428639

[19] Bakibillah ASM, Kamal MAS, Tan CP, Hayakawa T, Imura J-I. Optimal eco-driving scheme for reducing energy consumption and carbon emissions on curved roads. Heliyon. 2023;10(1):e23586. doi: 10.1016/j.heliyon.2023.e23586 38173479 PMC10761797

[20] Bakibillah ASM, Kamal MAS, Tan CP, Hayakawa T, Imura J. Fuzzy-tuned model predictive control for dynamic eco-driving on hilly roads. Appl Soft Comput. 2021;99:106875. doi: 10.1016/j.asoc.2020.106875

[21] Ahmed MM, Ghasemzadeh A. The impacts of heavy rain on speed and headway behaviors: An investigation using the SHRP2 naturalistic driving study data. Transp Res Part C: Emerg Technol. 2018;91:371–84.

[22] Ghasemzadeh A, Hammit BE, Ahmed MM, Young RK. Parametric ordinal logistic regression and non-parametric decision tree approaches for assessing the impact of weather conditions on driver speed selection using naturalistic driving data. Transp Res Rec: J Transp Res Board. 2018;2672(12):137–47. doi: 10.1177/0361198118758035

[23] Yang G, Ahmed MM, Gaweesh S. Impact of variable speed limit in a connected vehicle environment on truck driver behavior under adverse weather conditions: driving simulator study. Transp Res Rec: J Transp Res Board. 2019;2673(7):132–42. doi: 10.1177/0361198119842111

[24] Wang K, Zhang W, Feng Z, Yu H, Wang C. Reasonable driving speed limits based on recognition time in a dynamic low-visibility environment related to fog-A driving simulator study. Accid Anal Prev. 2021;154:106060. doi: 10.1016/j.aap.2021.106060 33706024

[25] Zhao X, Xu W, Ma J, Li H, Chen Y, Rong J. Effects of connected vehicle-based variable speed limit under different foggy conditions based on simulated driving. Accid Anal Prev. 2019;128:206–16. doi: 10.1016/j.aap.2019.04.020 31055185

[26] Gao K, Tu H, Sun L, Sze NN, Song Z, Shi H. Impacts of reduced visibility under hazy weather condition on collision risk and car-following behavior: Implications for traffic control and management. Int J Sustain Transp. 2019;14(8):635–42. doi: 10.1080/15568318.2019.1597226

[27] Yasanthi RGN, Mehran B. Modeling free-flow speed variations under adverse road-weather conditions: Case of cold region highways. Case Stud Transp Policy. 2020;8(1):22–30. doi: 10.1016/j.cstp.2020.01.003

[28] Gaweesh SM, Ahmed MM. Evaluating the safety effectiveness of a weather-based variable speed limit for a rural mountainous freeway in Wyoming. J Transp Safety Security. 2019;12(10):1205–30. doi: 10.1080/19439962.2019.1583707

[29] Das S, Geedipally SR, Fitzpatrick K. Inclusion of speed and weather measures in safety performance functions for rural roadways. IATSS Res. 2021;45(1):60–9. doi: 10.1016/j.iatssr.2020.05.001

[30] Romanowska A, Budzyński M. Investigating the impact of weather conditions and time of day on traffic flow characteristics. Weather Climate Soc. 2022;14(3):823–33. doi: 10.1175/wcas-d-22-0012.1

[31] Yasanthi RGN, Mehran B, Alhajyaseen WKM. A reliability-based weather-responsive variable speed limit system to improve the safety of rural highways. Accid Anal Prev. 2022;177:106831. doi: 10.1016/j.aap.2022.106831 36113332

[32] Guo J, Ma C, Xu X, Zhao Y, Lu X. Investigation on variable speed limit control strategy of expressway under adverse weather conditions. Physica A: Stat Mech Applic. 2022;602:127616. doi: 10.1016/j.physa.2022.127616

